# The Urgent Need for Dental Clinics

**Published:** 1912-02-17

**Authors:** 


### THE SCHOOL CLINIC.

### The Urgent Need for Dental Clinics.

Since 1907 a new work for the medical profession has been gradually coming into being. The organisation of the inspection of school children is now fairly complete in London, and the question of treatment is being considered. But there is one branch of disease which has as yet been hardly touched. We refer to dental caries in children. And yet this is probably the most important of all the classes of cases referred by the school doctor. The very reason for not tackling it has been its vastness; and that alone should show its importance.

### Their National Importance.

It is probable that treatment of dental caries would pay the nation better than treatment of all other diseases. The aim of national treatment should be preservative rather than curative. It is for the nation to put right any ills which may cause disease in the future rather than to keep alive by drugs and treatment those who are already inefficient. Caries of the teeth essentially comes under the former category. The number of children in whom the molars which erupted at six years are already carious by the age of ten or fourteen is simply appalling. If those children are left unattended the later teeth also become involved. Such carious teeth are often associated with a general oral sepsis, and the far reaching result of such conditions is even now but partially recognised.

In a former article we have pointed out the impossibility of treating children with disease of the throat unless the teeth are attended to at the same time; it is the same thing with dyspeptic conditions, and that general lowering of the health which it must be the aim of national treatment to improve. We may summarise our argument thus:—

(*a*) Treatment of dental conditions is as yet practically untouched.

(*b*) It will involve a larger percentage of children than any of the diseases which are being treated.

(*c*) The expense will be correspondingly greater.

(*d*) But the results of treatment of these cases should also show a greater increase of efficiency than in the other cases, and therefore the expense will be justified by the result.

(*e*) Much of the work now done in trying to treat other conditions while ignoring the mouth is equivalent to so much money thrown away.

It is a recognised fact that if everyone went to the dentist once in six months for examination the dentist's work would be very appreciably diminished. The ideal, therefore, would be to have every child's mouth inspected twice a year by a dentist, who would then decide on what treatment is necessary. But as the children are already once inspected, and as this would again add to the expense, we may consider this to be out of the range of practical politics. The medical inspector can recognise that the teeth need attention, though he may not be able to state whether they should be removed or treated conservatively.

All such cases should, therefore, be referred to a dentist for further consideration. The treatment which he will adopt will come under three heads—(1) Extraction, (2) conservative dentistry, (3) instruction in the care of teeth. Of these by far the most important is the third heading, a very small minority of the lower classes, and even of the lower middle-class, make use of the tooth-brush, and almost without exception their cure of toothache is to have the offending tooth removed. Of the other two it is probable that extractions will take a larger place than in the case of adults, decay of the milk teeth often only occurs but a short time before they are due to fall out of their own accord, and the treatment consists in their removal to prevent the involvement of the permanent set rather than to afford a longer life to the tooth itself. In the case of the permanent teeth it is important to treat any factor that may give rise to caries rather than to treat the condition when it has arisen.

We see, therefore, that the ultimate aim of any national treatment of dental caries must be to take the cases young, rather than to try to patch up the carious teeth of older children. No doubt there are many children in the schools who now need such treatment, but it is the younger ones who must particularly be attended to, and as time goes on the number of older children needing treatment should be considerably diminished.

### Organised Instruction.

This instructional treatment will need considerable care and thought, and no doubt some experimentation before it can be efficiently organised. It will not be hard to set up dental clinics either in every school or at some central spot for many schools, but to do this without teaching the children the value of the care of teeth would be a great waste of money. We have already pointed out that it is the younger children who must be seen to. It might be possible to have among the junior standards classes in ablutions and mouth-washing. We admit that many a child would pay no attention to them, and that the lessons learnt would be forgotten as soon as they passed into a higher standard. In a few, however, the lessons learnt may come back to them when adult life is attained, and they have children of their own to attend to. This brings us to the fact that it is the parents who must be taught rather than the children, and they must be taught young before they become parents. The ways of a large class of the population cannot be changed by an Act of Parliament, and it will take a generation before any very appreciable result can be seen.

We may sum up once more by saying that though the institution of dental clinics is urgently needed, it is only by the education of the children to take care of their own teeth, and of their children's when they become parents that any lasting benefit can be obtained in the health of the nation.

